# First report of filarial nematodes in the genus *Onchocerca* infecting black flies (Diptera: Simuliidae) in Iran

**DOI:** 10.1038/s41598-023-41890-z

**Published:** 2023-09-04

**Authors:** Fariba Khanzadeh, Naseh Maleki-Ravasan, Peter H. Adler, Fateh Karimian, Matus Kudela

**Affiliations:** 1 Department of Zoology, Comenius University, Bratislava, Slovakia; 2 Department of Parasitology, Pasteur Institute of Iran, Tehran, Iran; 3 Department of Plant and Environmental Sciences, Clemson University, Clemson, SC USA

**Keywords:** Ecology, Climate sciences, Health care, Medical research, Risk factors, Zoology, Entomology, Diseases, Eye diseases, Infectious diseases, Skin diseases

## Abstract

Black flies are blood-sucking insects of public health importance, and they are effective vectors of pathogens and parasites, such as filarial nematodes of the genus *Onchocerca*. Our previous surveys have shown that individuals of *Simulium turgaicum* are annoying pests of humans and livestock in the Aras River Basin of Iran. In the present study, adult black flies of *S. turgaicum* were trapped from different ecotopes of five villages in Khoda-Afarin County, Iran. By using a sensitive nested PCR assay and targeting the nuclear *18S rDNA-ITS1* marker, filarial infections were found in 38 (1.89%) of 2005 black flies. Homology exploration of 360 bp of the sequences indicated that the filarial worms are members of the family Onchocercidae, with maximum alignment scores of 93–95%. Phylogenetic analysis showed that two Iranian *Onchocerca* isolates were clustered in the *O. fasciata*–*O. volvulus* lineage and were well separated from other filarial nematodes. Both the entomological evidence (empty abdomen of the specimens) and climatologic data (adequate accumulated degree days for development) suggest that the filarial DNA was probably that of infective larvae of vertebrates. This is the first report of an infection by *Onchocerca* species in *S. turgaicum* and the first record of onchocercids in black flies in Iran; however, more research is required to demonstrate transmission of these filarial worms by black flies in nature.

## Introduction

As small, robust flies adapted for repeated blood-sucking, black flies can be significant pests of humans, livestock, and wildlife, influencing nearly all outdoor activities^1^. They are found globally and their distribution is largely influenced by the availability of running water and vertebrate hosts, which are required for the development of the immature stages and egg maturation, respectively^1,2^. Black flies are zoophilic and exophilic and are especially annoying to grazing animals. Anthropophilic black flies make up about 10% of the total simuliid fauna in each zoogeographic region. Because of their short, stout mouthparts, black flies cannot bite through clothing, and tend to primarily bite in areas that are thinly haired or feathered^1^.

Many parasites and pathogens, including protozoa, filarial nematodes, and viruses, are transmitted by black flies to domestic and wild animals, as well as humans^1^. Perhaps the most important role of black flies as vectors is the transmission of the filarial nematode *Onchocerca volvulus*, which causes human onchocerciasis or river blindness^3^. The parasite is transmitted from human to human by at least 26 *Simulium* species in tropical Africa, Latin America, and Yemen^1,4^.

Other species of *Onchocerca* parasitize domestic and wild animals (e.g., cattle, camels, horses, wild boars, deer, and dogs) and secondarily can infect humans. Zoonotic onchocerciasis is considered an emerging human parasitic disease, necessitating multidisciplinary approaches such as “One Health Strategies”, to detect and control new cases in humans. *Onchocerca* species of domestic animals and wildlife (e.g., *O. lupi*, *O. dewittei japonica*, *O. jakutensis*, *O. gutturosa*, and *O. cervicalis*) have been reported in humans as hypodermic nodules and ophthalmic infections. A few cases of *O. lupi*, *O. dewittei japonica*, and *O. jakutensis* have been reported recently in humans, possibly indicating completion of the parasite's life cycle in a new host^5^.

Transmission and incubation of *Onchocerca* species are largely influenced by environmental factors, such as temperature and precipitation, which regulate the population sizes and activities of the *Simulium* vectors and also influence the incubation period for filaria in the vector^6,7^. As in any other poikilothermic organism^8,9^, development of *Onchocerca* into infective third stage larvae (L3) in black flies can be quantified by accumulating degree days (ADD) between the temperature thresholds throughout the transmission season. In fact, ADD represent the combination between chronological time and temperature^10^. ADD begin from a biofixed date, the date of the first occurrence of a certain biological event such as the first emergence or capture of a pest, which can help predict when a growth stage will be reached^11^. However, these thermal and biotic parameters are not entirely known for *Simulium*–*Onchocerca* associations.

Members of the *Simulium* subgenus *Wilhelmia* are significant pests of humans and livestock in the Old World^12^, such as along the Kizilirmak River in Turkey^13,14^ and the Zayandeh-rood^15^ and Aras rivers^16^ in Iran. One of the major pests of the subgenus, *S. turgaicum*, is distributed from Slovenia and Bosnia and Herzegovina westward to China^17^. The pest status of *S. turgaicum* and other members of the group are related to their tendency to feed on large mammals and to achieve large populations^15^. The vectorial role of *S. turgaicum* has recently been investigated for zoonotic pathogens (e.g., *Dirofilaria* spp.)^2^. In the present study, *S. turgaicum* is molecularly screened for filarial infections in the Aras River Basin of Iran, and entomological and climatological evidence is provided that suggests it might be a vector of *Onchocerca*.

## Methods

### Study sites, sampling, and identification of black flies

This study was performed at the south of the Aras River in Khoda-Afarin County, East Azerbaijan Province, in northwestern Iran. Adult black flies were caught every two weeks from June to September 2017–2019, using a conventional aerial insect net, from four ecotopes (humans and animals outdoors, irrigation canals in rural areas, lands along the Aras River, and rice and alfalfa fields) in 5 villages (Deim-Qarloujeh, Gungormaz, Khetay, Larijan, and Qarloujeh). After being killed in a cyanide jar (Video S1), the insects were fixed in 70% ethanol at 20 °C until examination. The black flies were morphologically identified at the species level using the key by Crosskey^18^. Only specimens of *S. turgaicum* without visible blood-meals were used for filarial genome screening. Field observations of the bionomics and nuisance caused by *S. turgaicum* were documented during sampling or field trips through photography, videography, and interviews with local individuals. Three-second repeating videos were recorded with the help of the Boomerang program.

### DNA extraction, microfilariae detection, and bioinformatics analysis

All collected specimens of *S. turgaicum* with empty abdomens were subjected to filarial genome detection. They were first individually rinsed with 70% ethanol, and after dehydration, the whole bodies were ground in 1.5 ml microtubes. Total genomic DNA was extracted using the method of Collins et al.^19^. Filarial infections of *S. turgaicum* were investigated using a PCR assay by targeting the nuclear *18S rDNA-ITS1* marker used by Tang et al.^20^ as a sensitive and specific nested PCR for differentiating the major sympatric species of filaria in the Amazon Region. Their primers yielded amplicon sizes of 286–344 bp, depending on the species examined: 286 bp for *Loa loa*, 301 bp for *Wuchereria bancrofti*, 305 bp for *Mansonella ozzardi*, 312 bp for *Mansonella perstans*, 344 bp for *O. volvulus*, and 420 bp for *Dirofilaria* sp.

The reaction mixtures and PCR thermal cycling steps were adjusted based on previous studies^2^. PCR products were visualized with 2% agarose gel electrophoresis, followed by GreenView™ DNA Gel staining and photographing with a UV transilluminator. Two amplicons were randomly selected and sequenced using forward and reverse primers of nest-two by Genomin Company (Tehran, Iran). The sequences were deposited in GenBank under accession numbers ON342797 and MT052017. Twenty reference sequences belonging to numerous nematodes were retrieved from GenBank and used for multiple sequence alignment and phylogenetic analysis by MEGA X software^21^. Phylogenetic relationships were examined by maximum likelihood inference method. Confidence of internal nodes was confirmed by bootstrap analysis with 1000 replications. A sequence of target genes in spiruroid eyeworm, *Thelazia lacrymalis* (Accession Number: AY208137) was set as outgroup.

### Analysis of climatic parameters

The daily records of temperature and precipitation for 2011–2021 were obtained from the Khoda-Afarin meteorological station. Temperatures between the lower (16 °C) and upper (30 °C) developmental thresholds of *O. volvulus*^7^ were included for ADD calculations for each day. The ADD generated and required for the development of *Onchocerca* nematodes were calculated using a degree day calculator embedded in the UC-IPM Website and single sine model and horizontal cut-off method. To calculate the actual ADD required for *Onchocerca* microfilariae development, experimental data on *O. volvulus* development in specific simuliid vectors (including minimum and maximum laboratory temperatures and holding time of infected black flies) were extracted from the literature and submitted to the software with the same assumptions as above. To estimate the generated ADD, the minimum and maximum daily temperatures recorded by the Khoda-Afrin station were submitted to the software, taking into account the minimum and maximum developmental temperatures of *O. volvulus* and the above-mentioned defaults. The monthly degree days were used as the basis for computation of the possibility of *Onchocerca* species development throughout the year in the region. The empirical-objective correlation of different entomological, parasitological, and meteorological parameters was investigated by a diagram with GraphPad Prism.

## Results

### Entomological and parasitological findings

**Figure 1:**
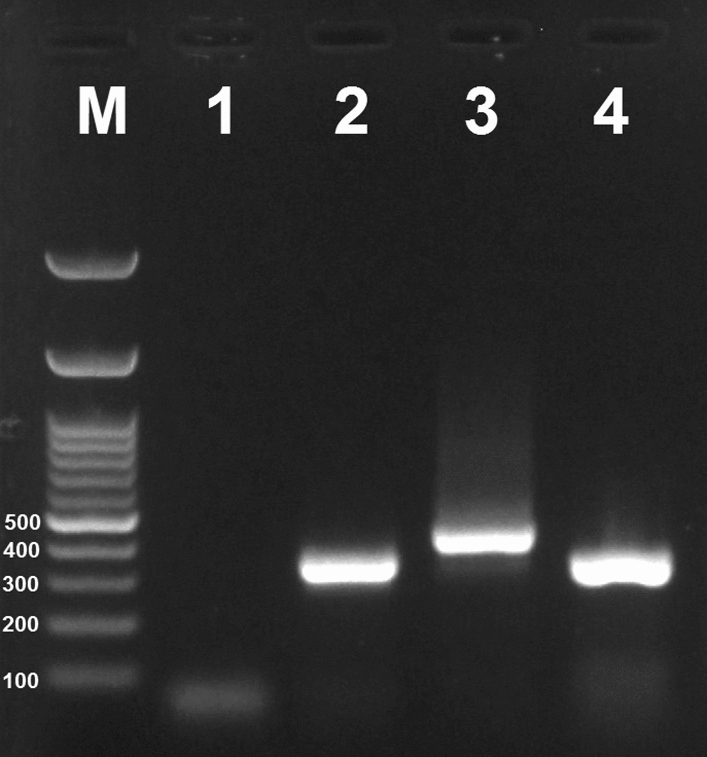
Species-specific nested PCR of filarial nematodes, using the *18S rDNA-ITS1* gene. Lanes: M, 100-bp ladder (Fermentas); 1, negative control; 2, 4, *Onchocerca* sp. (~ 350 bp); 3, *Dirofilaria* sp. as positive control (~ 420 bp).

**Figure 2:**
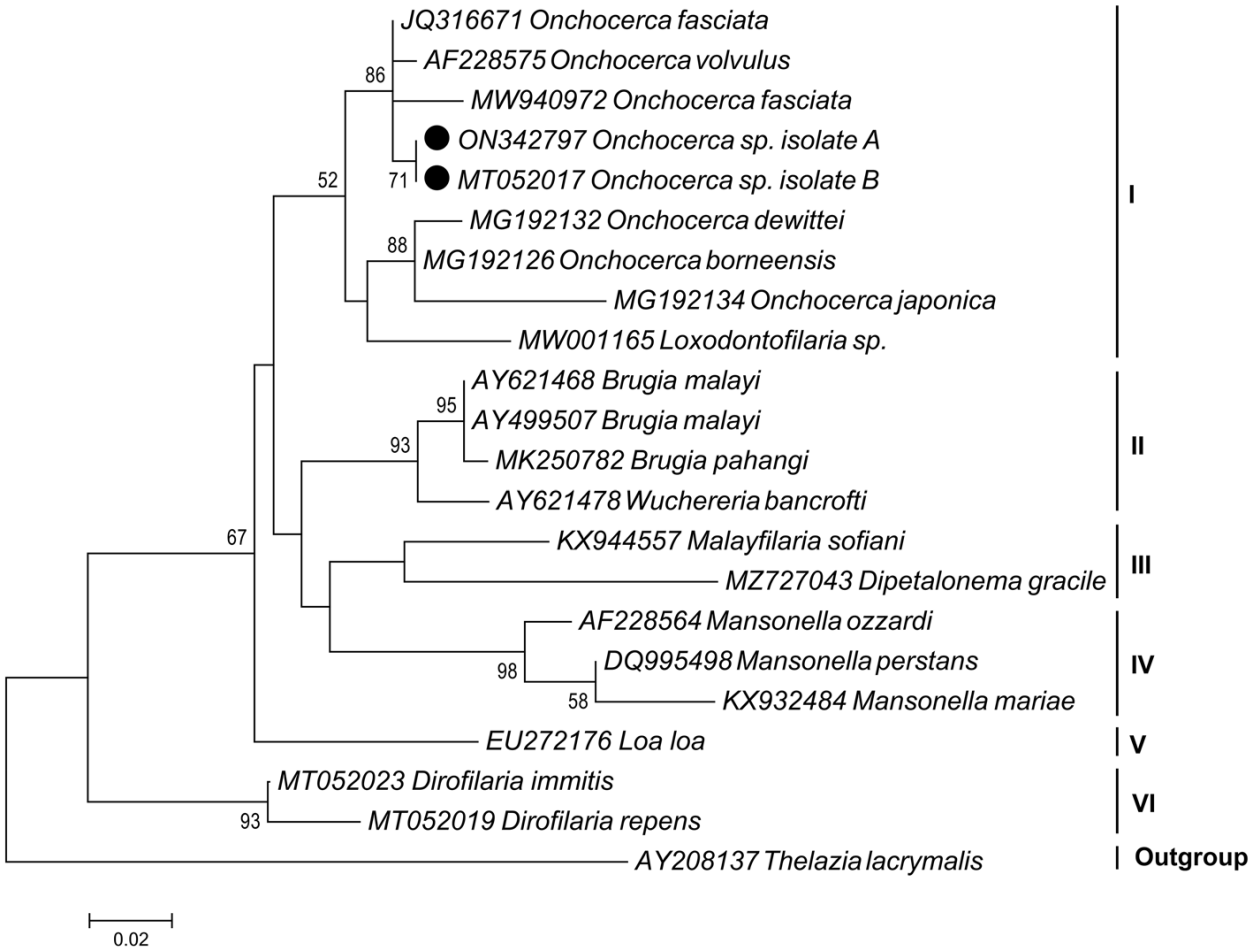
Maximum likelihood phylogenetic tree displaying the position of *Onchocerca* species identified in this study (solid circles) among other Onchocercidae filarial *18S rDNA-ITS1* sequences retrieved from GenBank. *Thelazia lacrymalis* was set as the outgroup. Numbers at the branch points are bootstrap values based on 1000 replicates. Bootstrap values less than 50% are not shown. The scale-bar measures evolutionary distance in substitutions per nucleotide.

A total of 2005 individuals of *S. turgaicum* were caught in different ecotopes in the villages of Khoda-Afarin County during three consecutive years. The highest and lowest numbers of specimens were collected in Qarloujeh (*n* = 795) and Khetay (*n* = 82), respectively. The most black flies were caught around humans and domestic animals outdoors (*n* = 1090) and the least around irrigation canals in rural areas (*n* = 260). The most *S. turgaicum* were caught in July (*n* = 781) and the least in June (*n* = 242) (Table 1). Filarial infections were detected in 38 (1.89%) of 2005 black flies (Table 1). For all positive specimens, the nested PCR assay resulted in a product size of ~ 350 bp (Fig. 1). The most and the fewest positive filarial specimens were captured around humans and animals outdoors (*n* = 18) and the lands along the Aras River (*n* = 3), respectively (Table 1). Homology exploration of 360 bp of two sequences obtained in our study (ON342797 and MT052017) showed that they belong to filarial nematodes of the family Onchocercidae, with maximum alignment scores of 93–95% (Table 2). The maximum likelihood analysis yielded a monophyletic tree with six groups (Fig. 2). There were 9 taxa clustered in Clade I, where all species, except *Loxodontofilaria* sp., were *Onchocerca* parasites. The Clade was divided into two subclades: the first included one *O. volvulus*, two *O. fasciata* along with two sequences of current study, and the second included the remaining four species. All Clades was supported by a strong bootstrap value (52–98), suggesting that two Iranian *Onchocerca* isolates is more closely related to *O. fasciata*–*O. volvulus* species than to the other species (Fig. 2).

**Table 1:** Females of *Simulium turgaicum* caught from June to August 2017–2019 in different ecotopes of the Aras River Basin, Iran, and tested for microfilariae infection.

Village	Ecotope	No. specimens used for filarial detection	No. infected (%)	Date of collection of infected flies*	Filariae species (Genbank ID)** and their ecotopes
Deim-Qarloujeh	Irrigation canals in rural areas (n = 222) Humans and animals outdoors (n = 147)	369	5 (1.35)	June 25, 2017 June 28, 2017 August 12, 2018 **July 11, 2019** July 30, 2019	*Onchocerca* sp. (ON342797) Humans and animals outdoors (n = 2), Irrigation canals in rural areas (n = 3)
Gungormaz	Lands along the Aras River (n = 86) Humans and animals outdoors (n = 125) Rice and alfalfa fields (n = 113)	324	9 (2.78)	June 30, 2017 July 8, 2017 August 2, 2018 August 5, 2018 August 18, 2018 August 19, 2018 June 26, 2019 July 30, 2019 August 8, 2019	*Onchocerca* sp. (NA) Humans and animals outdoors (n = 4), Rice and alfalfa fields (n = 5)
Khetay	Irrigation canals in rural areas (n = 28) Humans and animals outdoors (n = 54)	82	3 (3.66)	July 12, 2017 August 9, 2018 August 14, 2018	*Onchocerca* sp. (NA) Humans and animals outdoors (n = 2), Irrigation canals in rural areas (n = 1)
Larijan	Lands along the Aras River (n = 170) Humans and animals outdoors (n = 265)	435	4 (0.92)	June 22, 2017 August 23, 2017 June 26, 2019 July 20, 2019	*Onchocerca* sp. (NA) Humans and animals outdoors (n = 1), Lands along the Aras River (n = 3)
Qarloujeh	Rice and alfalfa fields (n = 296) Humans and animals outdoors (n = 499)	795	17 (2.14)	July 1, 2017 August 3, 2017 August 19, 2017 **June 30, 2018** August 1, 2018 August 6, 2018 August 15, 2018 August 20, 2018 September 1, 2018 June 24, 2019 June 27, 2019 July 14, 2019 July 17, 2019 July 20, 2019 July 31, 2019 August 5, 2019 August 7, 2019	*Onchocerca* sp. (MT052017) Humans and animals outdoors (n = 9), Rice and alfalfa fields (n = 8)
Total		2005	38 (1.89)		–-

*The dates of sequenced specimens are shown in bold.

**NA (not applicable) was used for specimens that were not sequenced and whose identity was determined by amplicon size.

**Table 2:** Megablast results for Onchocercidae *18S rDNA-ITS1* sequences obtained from *Simulium turgaicum* and details on the origin and location of isolates matching these queries.

Query sequence	Match	Identity	GenBank accession	Isolation source	Country
Onchocercidae filarial parasite (360 bp) isolate A (MT052017)	*Onchocerca fasciata*	342/361 (95%)	JQ316671	Camels (*Camelus bactrianus*)	China
Onchocercidae filarial parasite (314 bp) isolate B (ON342797)	*Onchocerca fasciata*	337/361 (93%)	MW940972	Camels (*Camelus dromedaries*)	Egypt
	*Onchocerca volvulus*	339/365 (93%)	AF228571	Humans (*Homo sapiens*)	Cameroon
	*Onchocerca volvulus*	308/332 (93%)	AF228574	Humans (*Homo sapiens*)	Cameroon
	*Onchocerca volvulus*	307/332 (92%)	AF228575	Humans (*Homo sapiens*)	Cameroon
	*Onchocerca volvulus*	307/332 (92%)	AF228576	Humans (*Homo sapiens*)	Cameroon
	*Onchocerca volvulus*	306/332 (92%)	AF228573	Humans (*Homo sapiens*)	Cameroon
	*Onchocerca volvulus*	308/336 (92%)	EU272179	Humans (*Homo sapiens*)	Brazil
	*Onchocerca volvulus*	308/336 (92%)	AF228572	Humans (*Homo sapiens*)	Cameroon
	*Onchocerca volvulus*	308/336 (92%)	AF228570	Humans (Homo sapiens)	Cameroon
	*Onchocerca volvulus*	308/336 (92%)	AF228568	Humans (*Homo sapiens*)	Brazil
	*Loxodontofilaria sp.*	327/373 (88%)	MW001165	Asian elephant (*Elephas maximus*)	Thailand
	*Onchocerca japonica*	298/345 (86%)	MG192134	Japanese boar (*Sus scrofa leucomystax*)	Japan
	*Onchocerca borneensis*	308/359 (86%)	MG192126	Bornean bearded pig (*Sus barbatus*)	Malaysia
	*Onchocerca borneensis*	295/344 (86%)	MG192125	Bornean bearded pig (*Sus barbatus*)	Malaysia
	*Onchocerca dewittei*	304/359 (85%)	MG192132	Banded pig (*Sus scrofa vittatus*)	Malaysia
	*Onchocerca dewittei*	293/344 (85%)	MG192130	Banded pig (*Sus scrofa vittatus*)	Malaysia

### Phenological assessment

**Figure 3:**
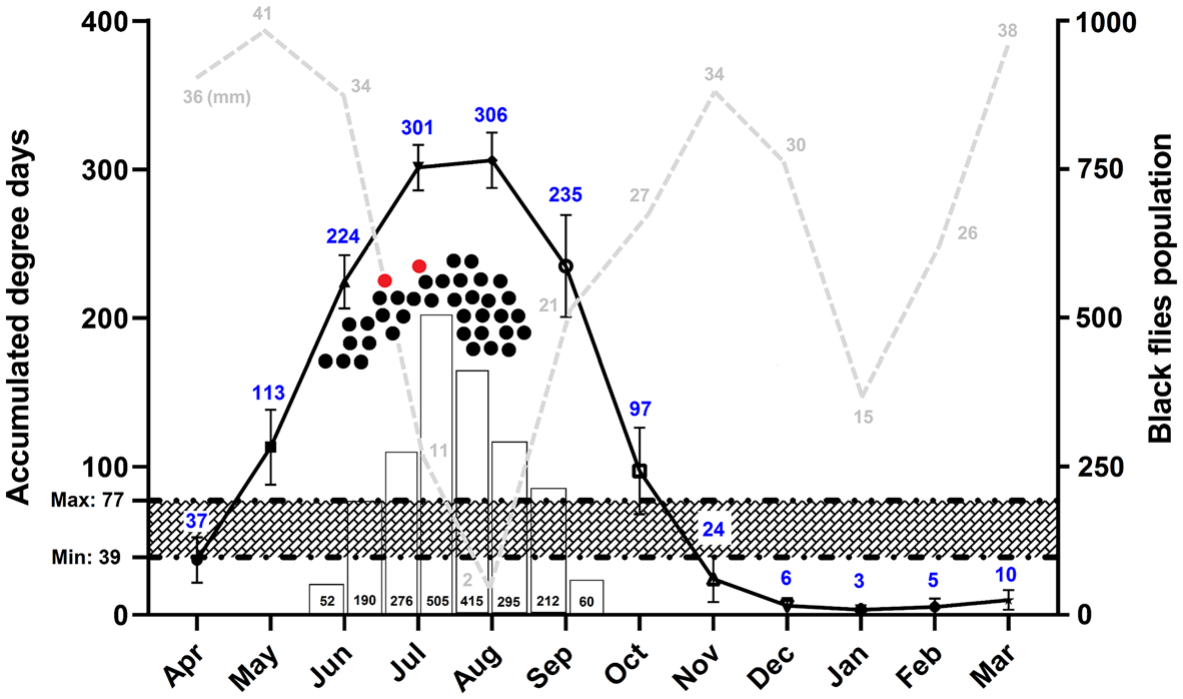
The associated role of environmental cues for development of *Onchocerca* microfilariae in *Simulium turgaicum* in Khoda-Afarin County, East Azerbaijan Province, Iran, 2011–2021. The relationship between the accumulated degree days (ADD) generated (upper curve) and required (hatched area) for the development of the nematodes, rainfall (gray lines), and fluctuations of the black fly population (bar graph) are shown. Temperatures between the lower (16 °C) and upper (30 °C) developmental thresholds were included for ADD calculations for each day. Black and red circles represent the dates of capture of *S. turgaicum* with microfilarial infections.

The calculation of ADD based on the data from 19 studies (Table S1) showed that an average of 58.23 (38.8–77.05) degree days is necessary for the development of *O. volvulus* in specific black fly vectors. While under natural conditions and taking into account the developmental thresholds of *O. volvulus*, 1313 degree days were generated for the Khoda-Afrin region, from April to October which could be up taken by microfilariae (Fig. 3). By considering the developmental thresholds of *O. volvulus*, the average ADD for 11 years was 1362.19 degree days. The average annual rainfall over 11 years was 113.51 mm, and the highest and lowest were in May (41 mm) and August (2.79 mm), respectively (Table S2). DNA of *Onchocerca* specimens was detected at the peak of *S. turgaicum* activity in the region, coinciding with the maximum ADD production—well above the average ADD required for the development of *O. volvulus* microfilariae under laboratory conditions—and minimum rate of rainfall (Fig. 3).

### Activity of *S. turgaicum*

*Simulium turgaicum* was active in the region during the early (6:00–11:00 a.m.) and late (17:00–19:00 p.m.) hours of each day from June to September. Although black flies are strong fliers, their activity was affected not only by humidity and temperature, but also by wind, so that on days without wind, 50–100 flies were caught per net, but on windy days, fewer than 10 were collected. Furthermore, natural and artificial lights were highly attractive to these flies and affected their activity (Videos S2, S3).

Based on our observations, *S. turgaicum* mainly attacked the head and sometimes the legs of humans, the head, tail, and legs of goats and sheep, the head and sometimes the tail of cattle, and the heads of dogs. Cattle tried to repel black flies by moving their ears, blinking, and moving their heads (Video S4). Large populations of black flies were observed in sheep manure storage areas and a handful (4–6) of black flies was observed indoors in human structures and near the larval habitat in late March.

The natives of Khoda-Afarin region consider black flies the most annoying group of insects. Black flies cause pain and itching of the ears and eyes and severe sneezing and coughing if they enter the mouth or nose, as confirmed in multiple face-to-face interviews with at least 25 people.

A 56-year-old farmer who came to his farm without a hat was bitten by a large number of black flies in his ears. He suffered inflammation of the ears, then pain and itching during the night; the inflammation and swelling continued for two days.

A 50-year-old woman was attacked by *S. turgaicum* in her ear while working in her garden next to a river. The fly was removed in the traditional way by using yellow oil, but she endured pain and itching of the ear for a prolonged time. After 5–6 months she was visited by a physician and treated with antibiotics. During this time, a nodule formed under the ear pinnae. By gentle rubbing, dried pus was pushed out and finally the pain subsided.

Two females, 13 and 45 years old, were bitten by black flies around their eyes, and went to a general practitioner with symptoms of inflammation and itching. Both cases improved after receiving sterile eye drops and completing the course of treatment.

## Discussion

*Simulium turgaicum* can have important economic and public health consequences over a wide geographic range, from Western Europe through the Middle East to East Asia, because of its high relative abundance and the problems it causes for humans and livestock. We report, for the first time, infections with *Onchocerca* species in *S. turgaicum*, adding possible epidemiological importance to its pest status. We also provide evidence that the DNA sequences found were likely related to infective larvae of vertebrates.

Our findings suggest that the causative agent of human or animal onchocerciasis is circulating in the area. However, for black flies to be incriminated as vectors of *Onchocerca* species, the L3 worms must be found in the head of the female flies^1^. If DNA of the filarial nematodes was found in blood-fed flies, it could be inferred that the flies acquired the worms by feeding from reservoir hosts. However, detection of filarial DNA in specimens with their abdomens empty of visible blood suggests that the parasite probably developed to the infectious L3 stage. As supportive data, DNA of the head, thorax and abdomen of a large number of S. *turgaicum* were separately examined for filarial infection, with the highest infection rates found in the head, abdomen and thorax, respectively (unpublished data).

Our search for filarial infections in *S. turgaicum* was initially based on the size of the PCR product and finally sequencing of two positive specimens. Although both methods have their own relative value, the results of the study could be strengthened by increasing the number of sequenced specimens and by using other identification markers. The species identity of the microfilariae could not be determined, either because similar sequences were absent from GenBank or the species is unknown. Our two *Onchocerca* sequences were clustered in the *O. fasciata*–*O. volvulus* lineage. *Onchocerca fasciata*, a subcutaneous filarial nematode, is the most common *Onchocerca* species in camelids^22^, and the pathology has been studied in detail in camels from central and southern Iran^23^. In Asia, Yemen is the only country where *Onchocerca volvulus* is known; the associated disease, human onchocerciasis, mainly affects rural communities near streams in western governorates^24^. *Simulium turgaicum* has multiple hosts, including humans, dogs, bovids, and birds; the majority of specimens (ca. 70%) feed on humans and/or dogs in Iran’s Aras River Basin, and 20% of the specimens show mixed dog and human blood in their guts^2^. Future studies, in addition to determining the species identity of the microfilariae in our study, should focus on the anthroponotic or zoonotic origin of these parasites and their clinical symptoms in animals and humans. In Iran, for example, a case of human ocular onchocerciasis caused by *O. lupi* has been reported from the center of the country^25^.

After being ingested by black flies, development of the microfilariae is temperature dependent to the L3 stage. *Onchocerca volvulus* can reach its infectious stage in different *Simulium* species at a temperature of 16.3–30.5 °C within 5–13 days^7^. Accordingly, the ADD required and generated for nematode development were calculated as 58 and 1313 degree days, respectively, which seems sufficient for the development of the unknown *Onchocerca* species in *S. turgaicum*. Thus, *S. turgaicum* has the potential to support larval development of the parasite to the infective stage in nature. The entomological, parasitological, and phenological factors during July and August are favorable to the occurrence of onchocerciasis in the region (Fig. 3). In these months, the population of *S. turgaicum*, nematode infection rate, and ADD are highest and rainfall in the region is lowest. At temperatures above 25°C, parasite development takes less than 7 days and, assuming a 3.5 day gonotrophic cycle and infection in the first bite, the fly can transmit L3 in its second gonotrophic cycle^7^. At lower temperatures, however, the transition occurs in the third blood feeding when the fly is about 8–10 days old^1^. Although each species needs a certain number of degree days to complete its growth, the values computed in our study provide estimates for the temperature requirements of the *Onchocerca* species to develop in *Simulium turgaicum*. The thermal requirements of the vector also should be considered. Although thermal tolerances are available for some species of black flies^26^, no data are available for *S. turgaicum*. With our results and the subsequent discovery of the additional necessary information, an understanding of the *Simulium*–*Onchocerca* relationships in the Aras River Basin should permit control measures, when needed, for both the vector and the parasite.

## Conclusion

Although many species in the genus *Simulium* are putative vectors of *Onchocerca* spp., many of the vectors and parasites remain unknown. We identified an *Onchocerca* parasite from *S. turgaicum* and suggested the time that entomological, parasitological, and environmental factors are ideal for establishment of the disease cycle in the Aras River Basin. More research, however, is required to demonstrate transmission of this filarial nematode in nature by *S. turgaicum*.

### Supplementary Information

Supplementary Information 1.

Supplementary Video 1.

Supplementary Video 2.

Supplementary Video 3.

Supplementary Video 4.

## Data Availability

All data supporting the findings of this study are available within the paper and its Supplementary Information. Sequence data were deposited into the GenBank under accession number ON342797 and MT052017 and are available at the following URL: https://www.ncbi.nlm.nih.gov/nuccore/.
