# Supplementary material for: First report of filarial nematodes in the genus Onchocerca infecting black flies (Diptera: Simuliidae) in Iran
Source: Sci Rep. 2023 Sep 4;13:14585. doi: 10.1038/s41598-023-41890-z (PMC10477325; doi:10.1038/s41598-023-41890-z)
Supplement: Supplementary file 1 — Supplementary Information 1. [file 41598_2023_41890_MOESM1_ESM.docx]

**Supplementary Table S.1**. The development times and temperature requirements of *Onchocerca volvulus* in various *Simulium* species.

| ***Simulium* species** | **Country** | **Temperature Range (°C)** | **Mean Temperature (°C)** | **Duration of development (days)** | **Accumulated Degree Days** | **Reference** |
| --- | --- | --- | --- | --- | --- | --- |
| *S. ochraceum* | Guatemala | 10 – 25 | 16.3 | 13.0 | 41.41 | 1 |
| *S. ochraceum* | Guatemala | 12 – 25 | 17.4 | 17.0 | 59.06 | 1 |
| *S. ochraceum* | Guatemala | 14 – 25 | 18.6 | 13.0 | 50.30 | 1 |
| *S. damnosum* | Burkina Faso | 12 – 26 | 19.0 | 10.8 | 43.30 | 2 |
| *S. ochraceum* | Guatemala | 16.1 – 25.6 | 19.4 | 8.0 | 38.80 | 3 |
| *S. metallicum* | Guatemala | 16.1 – 25.6 | 19.4 | 9.0 | 43.65 | 3 |
| *S. ochraceum* | Guatemala | 16 – 25 | 19.8 | 13.0 | 58.50 | 1 |
| *S. ochraceum* | Guatemala | 18 – 25 | 20.9 | 10.0 | 55 | 1 |
| *S. guianense* | Venezuela | 16 – 24.9 | 20.0 | 9.3 | 43.02 | 4 |
| *S. damnosum* | Cameroon | 19.5 – 23.5 | 21.5 | 7.9 | 44 | 5 |
| *S. soubrense & S. sanctipauli* | Cote D’Ivoire | 19 – 25 | 22.0 | 6.5 | 39 | 6 |
| *S. ochraceum* | Guatemala | 20 – 26 | 23.0 | 8.0 | 56 | 7 |
| *S. soubrense & S. sanctipauli* | Cote D’Ivoire | 18 – 30 | 24.0 | 6.5 | 52 | 8 |
| *S. soubrense & S. sanctipauli* | Cote D’Ivoire | 14 – 34 | 24.0 | 6.0 | 44.97 | 8 |
| *S. ochraceum* | Guatemala | 22 – 27 | 24.5 | 7.0 | 59.50 | 9 |
| *S. damnosum* | Tanzania | 23 – 26 | 24.5 | 6.0 | 51 | 10 |
| *S. ochraceum* | Guatemala | 24 – 26 | 25.0 | 7.7 | 69.3 | 11 |
| *S. damnosum* | Burkina Faso | 24 – 26 | 25.0 | 7.0 | 63 | 2 |
| *S. guianense* | Venezuela | 22 – 28 | 25.0 | 7.2 | 64.8 | 12 |
| *S. metallicum* | Venezuela | 22 – 28 | 25.0 | 5.5 | 49.5 | 4 |
| *S. damnosum* | Burkina Faso | 25 – 26 | 25.5 | 7.0 | 66.50 | 2 |
| *S. damnosum* | Burkina Faso | 24 – 27 | 25.5 | 6.8 | 62.7 | 2 |
| *S. damnosum* | Burkina Faso | 24 – 27 | 25.5 | 6.3 | 59.85 | 2 |
| *S. oyapockense* | Brazil | 24 – 27 | 25.5 | 7.0 | 66.5 | 13 |
| *S. damnosum* | Burkina Faso | 25 – 27 | 26.0 | 6.5 | 65 | 2 |
| *S. oyapockense* | Brazil | 24 – 28 | 26.0 | 7.0 | 70 | 13 |
| *S. limbatum/S. incrustatum* | Brazil | 24 – 28 | 26.0 | 6.0 | 60 | 13 |
| *S. damnosum* | Guinea? | 25 – 27 | 26.0 | 6.5 | 65 | 14 |
| *S. damnosum* | Burkina Faso | 26 – 27 | 26.5 | 6.8 | 71.4 | 2 |
| *S. metallicum* | Venezuela | 26 – 28 | 27.0 | 6.0 | 66 | 15 |
| *S. damnosum* | Nigeria | 26 – 28 | 27.0 | 6.0 | 66 | 16 |
| *S. damnosum* | Burkina Faso | 26 – 28 | 27.0 | 6.8 | 74.8 | 2 |
| *S. exiguum* | Ecuador | 25 – 30 | 27.5 | 6.7 | 77.05 | 17 |
| *S. oyapockense & S. incrustatum* | Venezuela | 27 – 30 | 28.5 | 5.8 | 72.50 | 18 |
| *S. exiguum* | Ecuador | 29 – 32 | 30.5 | 5.0 | 68.73 | 19 |
| Mean | --- | 20.84-27.04 | 16.3-30.5 | 7.84  (5-17) | 58.23  (38.8-77.05) | --- |

**Supplementary Table S.2.** The mean accumulated degree days (taking into account the developmental temperature thresholds of *Onchocerca volvulus*) and rainfall* in Khoda Afarin County, East Azerbaijan province, Iran during 2011-2021.

| Month  Year | Apr | May | Jun | Jul | Aug | Sep | Oct | Nov | Dec | Ran | Feb | Mar | Mean | Total |
| --- | --- | --- | --- | --- | --- | --- | --- | --- | --- | --- | --- | --- | --- | --- |
| 2011 | 40.01  (22.80) | 81.58  (59.40) | 216.78  (55.00) | 318.43  (1.40) | 325.91  (7.00) | 164.34  (44.00) | 83.46  (25.10) | 2.33  (86.70) | 0.33  (8.00) | 2.21  (15.00) | 0  (33.80) | 4.75  (47.00) | 103.34  (33.77) | 1240.13  (405.2) |
| 2012 | 58.63  (12.00) | 122.91  (41.40) | 242.5  (47.20) | 273.31  (34.60) | 315.74  (0.00) | 223.76  (11.80) | 154.73  (0.00) | 46.75  (30.00) | 6.76  (25.00) | 0.23  (16.80) | 7.08  (44.00) | 12.45  (39.20) | 122.07  (25.17) | 1464.85  (302.00) |
| 2013 | 48.75  (31.60) | 116.35  (22.60) | 217.69  (46.00) | 297.4  (9.00) | 281.53  (0.00) | 256.29  (0.00) | 86.63  (25.40) | 10.41  (14.80) | 8.14  (51.00) | 0  (0.00) | 1.16  (1.70) | 8.72  (16.20) | 111.09  (18.19) | 1333.07  (218.30) |
| 2014 | 36.34  (20.80) | 153.3  (68.20) | 219.13  (139.20) | 304.95  (9.90) | 316.8  (0.00) | 275.08  (1.20) | 72.08  (65.60) | 15.07  (34.20) | 3.06  (80.20) | 2.88  (20.00) | 6.74  (32.40) | 2.21  (67.40) | 117.30  (44.85) | 1407.64  (538.20) |
| 2015 | 28.01  (70.60) | 59.58  (33.60) | 231.11  (1.80) | 311.42  (5.80) | 308.05  (3.80) | 231.85  (32.40) | 115.13  (31.60) | 17.26  (64.80) | 11.76  (2.20) | 6.99  (22.80) | 5.24  (1.80) | 16.81  (20.00) | 111.93  (24.26) | 1343.21  (291.2) |
| 2016 | 18.77  (80.00) | 111.62  (26.20) | 194.55  (9.20) | 281.57  (5.40) | 298.4  (12.00) | 232.18  (4.00) | 76.03  (32.14) | 9.92  (51.50) | 3.31  (12.60) | 0  (0.00) | 0.01  (35.60) | 4.06  (45.40) | 102.53  (26.17) | 1230.42  (314.04) |
| 2017 | 27.94  (25.80) | 113.72  (18.90) | 217.99  (21.40) | 300.01  (0.00) | 324.7  (0.00) | 279.5  (0.00) | 67.13  (66.40) | 38.55  (31.60) | 1.72  (22.60) | 3.38  (0.00) | 8.8  (31.60) | 22.12  (31.20) | 117.13  (20.79) | 1405.56  (249.50) |
| 2018 | 42.97  (17.00) | 118.82  (58.40) | 197.18  (53.80) | 324.4  (0.00) | 304.8  (0.00) | 246.35  (11.20) | 114.96  (10.00) | 34.54  (33.40) | 9.97  (29.20) | 3.85  (15.00) | 1.91  (61.00) | 10.94  (35.80) | 117.56  (27.07) | 1410.69  (324.80) |
| 2019 | 24.03  (62.60) | 119.4  (31.40) | 240.56  (0.00) | 293.13  (5.40) | 306.53  (0.00) | 187.27  (83.20) | 113.32  (0.00) | 34.77  (1.40) | 5.27  (32.60) | 8.51  (31.20) | 6.17  (14.00) | 18.11  (38.00) | 113.09  (24.98) | 1357.07  (299.80) |
| 2020 | 18.89  (45.60) | 106.16  (42.70) | 243.05  (2.20) | 294.46  (49.00) | 265.02  (2.40) | 236.75  (40.60) | 123.09  (34.40) | 42.33  (0.00) | 0  (56.80) | 8.23  (6.20) | 19.45  (32.20) | 5.45  (19.40) | 113.57  (27.62) | 1362.88  (331.50) |
| 2021 | 63.5  (10.00) | 139.67  (46.00) | 245.28  (0.00) | 313.15  (0.00) | 318.21  (0.00) | 248.95  (0.00) | 60.14  (6.60) | 13.51  (23.50) | 17.19  (5.60) | 1.16  (35.00) | 3.31  (0.00) | 4.5  (53.40) | 119.04  (15.01) | 1428.57  (180.10) |
| Mean | 37.08  (36.25) | 113.01  (40.80) | 224.17  (34.16) | 301.11  (10.87) | 305.97  (2.29) | 234.76  (20.76) | 96.97  (27.02) | 24.13  (33.81) | 6.14  (29.62) | 3.40  (14.73) | 5.44  (26.19) | 10.01  (37.54) | 113.51  (26.17) | 1362.19 |
| Total | 407.84  (398.80) | 1243.11  (448.80) | 2465.82  (375.80) | 3312.23  (119.60) | 3365.69  (25.20) | 2582.32  (228.40) | 1066.70  (297.24) | 256.44  (371.90) | 67.51  (325.80) | 37.44  (162.00) | 59.87  (228.10) | 110.12  (413) | 1248.67 | 14984.09  (3554.64) |

* The numbers in parentheses show the average monthly rainfall in mm.

**Supplementary Video S.1.** Rapid killing of captured *Simulium* spp. by a cyanide jar with minimal damage

**Supplementary Video S.2.** Black flies attraction to the setting sun light.

**Supplementary Video S.3.** Black flies attraction to artificial light.

**Supplementary Video S.4.** The cattle grooming behaviour to get rid of black flies.

**Supplementary Figure S.1.** The original and unprocessed gel picture showing species-specific nested-PCR of filarial nematodes, using the *18S rDNA-ITS1* gene. Lanes: M, 100-bp ladder (Fermentas); 1, negative control; 2, 4, *Onchocerca* sp. (~350 bp); 3, *Dirofilaria* sp. as positive control (~ 420 bp).


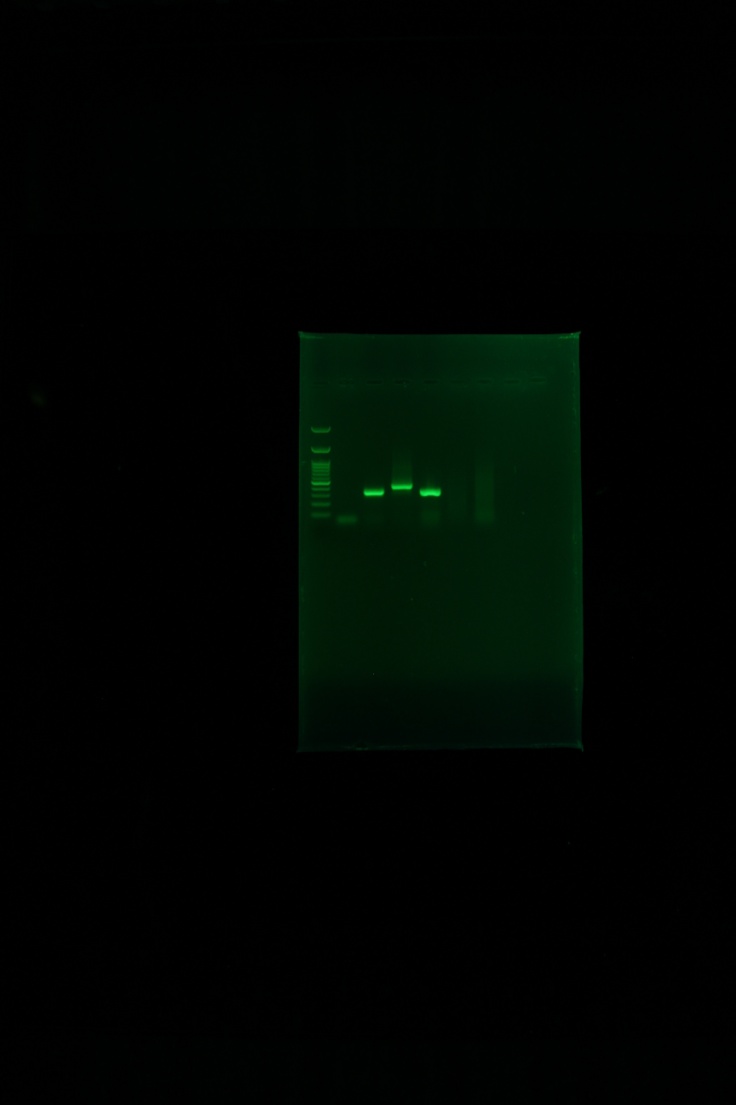


**References**

1 Takaoka, H., Ochoa, J. O., Juarez, E. L. & Hansen, K. M. Effects of temperature on development of *Onchocerca volvulus* in *Simulium ochraceum*, and longevity of the simuliid vector. *The Journal of Parasitology*, 478-483 (1982).

2 B., P. E´tude de la transmission *d’Onchocerca volvulus* (Leuckart, 1893) (Nematoda, Onchocercidae) par *Simulium damnosum* Theobald, 1903 (Diptera: Simuliidae) en Afrique tropicale. *Trav. Doc. O.R.S.T.O.M.* **63,** 308 (1977).

3 Collins, R. Development of *Onchocerca volvulus* in *Simulium ochraceum* and *Simulium metallicum*. *The American Journal of Tropical Medicine and Hygiene* **28**, 491-495 (1979).

4 Takaoka, H. *et al.* Development of *Onchocerca volvulus* larvae in *Simulium pintoi* in the Amazonas region of Venezuela. *The American journal of tropical medicine and hygiene* **33**, 414-419 (1984).

5 Eichner, M., Renz, A., Wahl, G. & Enyong, P. Development of *Onchocerca volvulus* microfilariae injected into *Simulium* species from Cameroon. *Medical and Veterinary Entomology* **5**, 293-298 (1991).

6 Prod’hon, J., Lardeux, F., Bain, O., Hébrard, G. & Prud’hom, J.-M. Ivermectine et modalités de la réduction de l’infection des Simulies dans un foyer forestier *d’onchocercose humaine*. *Annales de Parasitologie humaine et comparee* **62**, 590-598 (1987).

7 Collins, R., Campbell, C., Wilton, D. & Newton, L. Quantitative aspects of the infection of *Simulium ochraceum* by *Onchocerca volvulus*. *Tropenmedizin und Parasitologie* **28**, 235-243 (1977).

8 Prod'hon, J., Hébrard, G., Prud’hom, J.-M. & Couret, D. Etude de la capacité vectrice expérimentale de *Simulium soubrense*–*Simulium sanctipauli* en zone de savane humide (Région de Touba, Côte d’Ivoire). *Unpublished report, OCCGE/ORSTOM, Bouake, Côte d’Ivoire. Available at http://horizon. documentation. ird. fr/exldoc/pleins_textes/pleins_textes_5/b_fdi_02-03/02701. pdf (accessed 5th May 2014)* (1983).

9 De Leon, J. R. & Duke, B. Experimental studies on the transmission of Guatemalan and West African strains of *Onchocerca volvulus* by *Simulium ochraceum*, *S. metallicum* and *S. callidum*. *Transactions of the Royal Society of Tropical Medicine and Hygiene* **60**, 735-752 (1966).

10 Wegesa, P. The resettlement of refugees and onchocerciasis in Tanzania. *East African Medical Journal* **45**, 251-253 (1968).

11 Matsuo, K., Okazawa, T., Onishi, O. & Ochoa, A. Experimental observation of developmental period of *Onchocerca volvulus* in black fly, *Simulium ochraceum*. *Jap. J. Parasitol* **29**, 13-17 (1980).

12 Basáñez, M. G. *et al.* The vectoral role of several blackfly species (Diptera: Simuliidae) in relation to human onchocerciasis in the Sierra Parima and Upper Orinoco regions of Venezuela. *Annals of Tropical Medicine & Parasitology* **82**, 597-611 (1988).

13 Shelley, A., Dias, A. L., Moraes, M. & Procunier, W. The status of *Simulium oyapockense* and *S*. *limbatum* as vectors of human onchocerciasis in Brazilian Amazonia. *Medical and Veterinary Entomology* **1**, 219-234 (1987).

14 Bain, O. Morphologie des stades larvaires *d’Onchocerca volvulus* chez *Simulium damnosum* et redescription de la microfilaire. *Annales de Parasitologie humaine et comparée* **44**, 69-81 (1969).

15 Nelson, G. Human onchocerciasis: notes on the history, the parasite and the life cycle. *Annals of Tropical Medicine & Parasitology* **85**, 83-95 (1991).

16 Gemade, E. & Dipeolu, O. Onchocerciasis in the Benue State of Nigeria: II. Prevalence of the disease among the Tivs living in the Kwande Local Government area. *Annals of Tropical Medicine & Parasitology* **77**, 513-516 (1983).

17 Takaoka, H., Tada, I., Baba, M. & Shimada, M. Ecuador as the Vector of Human Onchocerciasis0. *Jpn. J. ParasitoL* **37**, 76-83 (1988).

18 Grillet, M.-E. *et al.* Vector competence of *Simulium oyapockense* sl and *S. incrustatum* for *Onchocerca volvulus*: implications for ivermectin-based control in the Amazonian focus of human onchocerciasis, a multi-vector–host system. *Acta tropica* **107**, 80-89 (2008).

19 Collins, R. C., Lehmann, T., JC, V. G. & Guderian, R. H. Vector competence of *Simulium exiguum* for *Onchocerca volvulus*: implications for the epidemiology of onchocerciasis. *The American journal of tropical medicine and hygiene* **52**, 213-218 (1995).
